# *Taraxacum officinale* and *Urtica dioica* extracts inhibit dengue virus serotype 2 replication in vitro

**DOI:** 10.1186/s12906-018-2163-3

**Published:** 2018-03-16

**Authors:** María R. Flores-Ocelotl, Nora H. Rosas-Murrieta, Diego A. Moreno, Verónica Vallejo-Ruiz, Julio Reyes-Leyva, Fabiola Domínguez, Gerardo Santos-López

**Affiliations:** 1 Laboratorio de Biología Molecular y Virología, Centro de Investigación Biomédica de Oriente, Instituto Mexicano del Seguro Social, Km 4.5 Carr Federal Atlixco-Metepec, 74360 Metepec, Puebla México; 2 Laboratorio de Biotecnología de Productos Naturales, Centro de Investigación Biomédica de Oriente, Instituto Mexicano del Seguro Social, Km 4.5 Carretera Federal Atlixco-Metepec, CP 74360 Metepec, Puebla México; 3 Posgrado en Ciencias Químicas, Benemérita Universidad Autónoma de Puebla, Ciudad Universitaria, Avenida San Claudio s/n, Puebla, 72570 México; 4 Laboratorio de Bioquímica, Centro de Química, Instituto de Ciencias, Benemérita Universidad Autónoma de Puebla, Avenida San Claudio s/n, Puebla, 72570 México; 5 Department of Food Science and Technology, Phytochemistry and Healthy Foods Lab, CEBAS-CSIC, Campus Universitario de Espinardo 25, E-30100 Murcia, Spain

**Keywords:** *Taraxacum officinale*, *Urtica dioica*, *Calea integrifolia*, *Caesalpinia pulcherrima*, Dengue virus, Antiviral

## Abstract

**Background:**

*Urtica dioica*, *Taraxacum officinale*, *Calea integrifolia* and *Caesalpinia pulcherrima* are widely used all over the world for treatment of different illnesses. In Mexico, these plants are traditionally used to alleviate or counteract rheumatism and inflammatory muscle diseases. In the present study we evaluated the activity of aqueous and methanolic extracts of these four plants, on the replication of dengue virus serotype 2 (DENV2).

**Methods:**

Extraction process was carried out in a Soxtherm® system at 60, 85 and 120 °C; a chemical fractionation in silica gel chromatography was performed and compounds present in the active fractions were identified by HPLC-DAD-ESI/MSn. The cytotoxic concentration and the inhibitory effect of extracts or fractions on the DENV2 replication were analyzed in the BHK-21 cell line (plaque forming assay). The half maximal inhibitory concentration (IC_50_) and the selectivity index (SI) were calculated for the extracts and fractions.

**Results:**

The methanolic extracts at 60 °C of *T. officinale* and *U. dioica* showed the higher inhibitory effects on DENV2 replication. After the chemical fractionation, the higher activity fraction was found for *U. dioica* and *T. officinale,* presenting IC_50_ values of 165.7 ± 3.85 and 126.1 ± 2.80 μg/ml, respectively; SI values were 5.59 and 6.01 for each fraction. The compounds present in *T. officinale*, were luteolin and caffeoylquinic acids derivatives and quercertin diclycosides. The compounds in the active fraction of *U. dioica*, were, chlorogenic acid, quercertin derivatives and flavonol glycosides (quercetin and kaempferol).

**Conclusions:**

Two fractions from *U. dioica* and *T. officinale* methanolic extracts with anti-dengue activity were found. The compounds present in both fractions were identified, several recognized molecules have demonstrated activity against other viral species. Subsequent biological analysis of the molecules, alone or in combination, contained in the extracts will be carried out to develop therapeutics against DENV2.

## Background

Dengue fever is a viral disease transmitted by mosquitoes in the genus *Aedes*, the principal species are *A. aegypti* and *A. albopictus*. The infection occurs mainly in tropical and subtropical regions of the planet. The number of cases in the past 30 years has increased considerably, this disease affects more than 100 countries around the world with 100 million cases each year, 500 thousand requiring hospitalization, and approximately 25,000 resulting in death each year [1–3].

Dengue virus (DENV), a member of the *Flaviviridae* family, is an enveloped virus containing a ~ 11 kb genome of positive single-stranded RNA which encodes three structural proteins (C, pr-M, E) and seven nonstructural proteins (NS1, NS2A, NS2B, NS3, NS4A, NS4B, NS5) [4]. Four serotypes of dengue virus (DENV1, DENV2, DENV3 and DENV4) cause dengue fever (DF) and more severe manifestations like dengue hemorrhagic fever (DHF) and dengue shock syndrome (DSS) [1].

Currently there are no specific antiviral compounds for the treatment, however, several research groups have sought antiviral compounds by molecular docking [5, 6] or using medicinal plants to inhibit a viral target [7, 8]. Current treatment to severe dengue is supportive fluid therapy under medical supervision [9]. Having no specific antiviral therapy or an antiviral agent for dengue treatment, different methods for prevention have been established by controlling the mosquito reproduction or spread [10, 11]. Due to the lack of new molecules, some clinical researches have proposed the repurposing of well-known drugs such as chloroquine, prednisolone, balapiravir, celgosivir, and lovastatin, however, although those drugs are safe, they have not been successful at decreasing viral load, antigenemia, fever or inducing a beneficial effect to dengue patients [12].

Ethnopharmacology has contributed significantly to the discovery of new drugs [13, 14]. In recent years, the focus on medicinal plants widely used in traditional systems has increased worldwide [15]. Based on ethnobotanical information some studies have demonstrated several compounds with anti-dengue potential activity such as, 7-0-methyl-glabranine [16], baicalein [17], catanospermine [7], quercetin and fisetin [18, 19].

Within the Mexican population, dengue is commonly known as “five-day fever”, “breakbone fever” or simply “breaker”. For the treatment of this disease there are therapeutic resources of traditional medicine, whose information has been collected over many years in important databases such as the Medicinal Herbarium of the Mexican Social Security Institute (Herbarium IMSS-M) [20]. In this collection, multiple uses of medicinal plants native to Mexico are referenced, including some that have traditionally been used against dengue, such as *Taraxacum officinale, Urtica dioica, Calea integrifolia* and *Caesalpinia pulcherrima.*

These plants are widely used in traditional medicine around the world for the treatment of many illnesses. *U. dioica* has great medicinal potential, its extracts have been used for the treatment of eczema, digestion, pain, anemia, arthritis, rheumatism [21], and it inhibits inflammatory processes caused by seasonal allergies [22]. *T. officinale* is used in the treatment of anemia, liver cirrhosis, rheumatoid arthritis and also it has been reported with anti-inflammatory, anti-oxidative, anti-carcinogenic, analgesic, anti-hyperglycemic, laxative and diuretic activities and also as stimulating for the digestive system [23]. It was reported that *T. officinale* has inhibitory potential against HIV and its reverse transcriptase [24].

*C. pulcherrima* is used in the treatment of cough [25], contains flavonoids and some reports show that aqueous extracts of flowers, leaves and stem, have inhibitory effect on several viruses, including herpes (HSV1-2) and adenoviruses (ADV-3, ADV-8, ADV-11) [26]. Meanwhile, *C. integrifolia* has been reported with antihyperglycemic activity and used in the treatment of diabetes [27, 28].

Of these plant species, only *U.dioica* has been reported as a source of an anti-dengue constituent, since the N-Acetyl-D-Glucosamine-specific lectin of this plant (UDA) has proved effective at reducing the viral infection of the four dengue serotypes [29]. However, other compounds, distinct from proteins have not been studied. Therefore, in this work it was analyzed if the aqueous and methanolic extracts of these four plants have inhibitory activity on DENV2 replication.

## Methods

### Reagents

Solvents methanol, ethyl acetate, formic acid and dimethyl sulfoxide (DMSO) reagent or HPLC grade and silica gel (Kiselgel 69) were obtained from Merck KGaA (Darmstadt, Germany). Media and supplements for cell culture were purchased from Sigma-Aldrich Chemicals (St. Louis, MO, USA) and molecular grade agarose was obtained from Promega (Madison, USA). All other reagents used in analytical methods were purchased from Sigma-Aldrich Chemicals, except where otherwise indicated.

### Cells

C6/36 cell line (*A. albopictus*) was maintained in minimum essential medium (MEM) supplemented with 5% fetal bovine serum (FBS), 100 U/mL penicillin, 100 μg/mL streptomycin. Cell line BHK-21 (hamster kidney neonate) was cultured in MEM supplemented with 5% FBS and antibiotics. Cells were incubated at 37 °C in 5% CO_2_. As is recommended for this type of experiments, *Aedes albopictus* C6/36 cells (ATCC: CRL-1660) were used for viral propagation [30] and baby hamster kidney BHK-21 cells (ATCC: CCL-10) were used to quantify the virus by plaque reducing assays [7, 31].

### Virus

Dengue virus serotype 2 (DENV2) strain Thailand/16681/1984 used in this study was kindly provided by Dr. Alvaro Aguilar-Setien (IMSS, Mexico City, Mexico). To obtain the inoculum used in all experiments, dengue virus was replicated in C6/36 cells for 5 days. Subsequently the viral supernatant was centrifuged for 5 min at 10,000 rpm to remove the cell debris. Aliquots were stored at − 70 °C until use [32, 33].

### Viral titration

Infection of the cells by DENV2 was confirmed by reverse transcription-polymerase chain reaction (RT-PCR) using reported specific primers [34] and by immunofluorescence using an antibody against the viral prM protein, as previously reported by our group [32, 33]. Quantification of DENV2 was performed by the lytic plaque assay in BHK-21 cells on six wells plates; after 24 h, the cells were washed with PBS and infected with 10-fold serial dilutions of the virus inoculum. After 1 h, the not absorbed virus was removed, cells were washed with PBS and then 0.35% agarose and DMEM were added. Plates were incubated for 72 h at 4 °C and 5% CO_2_. After that, 5% trichloroacetic acid was used to fix the cells and subsequently they were stained with 0.05% crystal violet in 20% ethanol [32, 33, 35].

### Selection and collection of medicinal plants

The plant species *T. officinale, U. dioica, C. integrifolia and C. pulcherrima* used in this study were identified as likely sources of active compounds against dengue virus from ethnobotanical information obtained from Herbarium IMSS-M (Catalogs: 1988, 1990, 1994; available in paper format only), which concerns its use in syndromes or diseases which are probably dengue, such as inflammatory muscle pain. All the plant specimens were collected in the state of Puebla, Mexico, in the municipalities of San Jeronimo Tecuanipan (19° 00′ 00″ North, 98° 24′ 54″ West) and in Atlixco (18° 54′ 45″ North, 98° 25′ 40″ West). The aerial part of the plants was cropped with contaminant-free cutter and transported to the laboratory. The vegetal material was taxonomically identified by experts of the Herbarium IMSS-M at Mexico City and reference vouchers of the plants material *T. officinale, U. dioica, C. integrifolia and C. pulcherrima* were deposited with the codes IMSS-M 164320, 164340, 17080 and 17120, respectively.

### Processing of plants and obtaining of crude extracts

Leaves of each plant were separated, washed and air-dried at room temperature (26 °C) for 2 weeks, after which they were grinded to a uniform powder in a blender (Nutribullet LLC, Pacoima, CA, USA). The extraction from pulverized plants was conducted in a Soxtherm® system (Gerhardt, Königswinter, Germany) at 60, 85 and 120 °C using methanol or water as solvents. In the extraction process the variables were the solvents and the extraction temperatures, but the extraction time (60 min), the reduction range (30 s) and the pulse reduction (2 s) remained constant. Methanolic and aqueous extracts were dried using a rotary evaporator (Heidolph Instruments Gmbh & Co. KG, Germany) at 60 °C or 90 °C, respectively. The dried residue was weighed, dissolved in DMSO [17] and used for cytotoxicity and inhibition of viral replication tests.

### Determination of cytotoxicity

The cytotoxic concentration 50 (CC_50_) was obtained in BHK-21 cells with the conventional MTT (3-(4,5-dimethylthiazol-2-yl)-2,5-diphenyltetrazolium bromide) colorimetric assay by serial dilutions (between 0.1 and 1200 μg/ml) of the extracts in DMSO, used as vehicle to all extracts or fractions. After 3 days of incubation at 37 °C and 5% CO_2_, the culture medium was removed, the cells were washed twice with PBS and the standard MTT protocol was performed [36]. Experiments were carried out in triplicate and done twice.

### Viral inhibition assays

BHK-21 cells were seeded in 6-wells plates during 24 h. The infection was performed with 0.5 ml of DENV inoculum in a dilution to produce approximately 100 lytic plaques per well (a 1:3100 dilution from a titrated viral inoculum at 3.1X10^6^ PFU/ml). After 1 h incubation, the viral inoculum was removed and washed with PBS twice. Immediately, different concentrations of each of the extracts were added. As control for inhibition the study used: a) cells without extract and without infection, b) infected cells without extract and, c) cells without infection and with extract [37]. Subsequently molecular biology grade agarose was added to a final concentration of 0.35% in DMEM with 2.5% FBS. After 3 days, the cells were fixed with 10% trichloroacetic acid for 10 min and stained with 0.1% crystal violet for 3 min. Results of the inhibition of viral replication were reported as a percentage of the formed lytic plaques compared to the control infection assay (100%), which was not treated with plant extracts [32, 33]. Experiments were carried out in triplicate and done twice.

### Chromatographic fractionation

*T. officinale* and *U. dioica* extracts were fractionated. The extracts were suspended in deionized water and partitioned twice with dichloromethane and the solvents were exhaustively removed to obtain dichloromethane fraction. This fraction was subjected to silica gel column (10 × 30 cm) and eluted with a gradient of n-hexane-ethyl acetate (9:1, 8:2,…1:1) to yield 17 fractions. These fractions were evaluated in the same biologic assays.

### Identification of compounds by HPLC-DAD-ESI/MSn

For the chemical analysis, 100 mg of sample were mixed with 1.5 mL of methanol:water:formic acid (25:24:1, v:v:v) extractant, then vortexed and sonicated in an ultrasonic bath for 60 min at room temperature. The samples were kept at 4 °C overnight and sonicated again for 60 min. A centrifugation was performed for 10 min at 10,000 rpm to separate the supernatant from the solid residue. The supernatant was filtered through a 0.22 μm PVDF filter before analysis (Millipore, MA, USA). The chromatographic analyses for identification of compounds were carried out on a Luna C18 column (250 × 4.6 mm, 5 μm particle size; Phenomenex, Macclesfield, UK). Water/formic acid (99:1, *v*/v) and acetonitrile were used as the mobile phases A and B, respectively, with a flow rate of 1 mL/min. The linear gradient started with 8% solvent B, reaching 15% solvent B at 25 min, 22% at 55 min, and 40% at 60 min, which was maintained to 70 min. The injection volume was 20 μL, and the analyses were carried out using an Agilent HPLC 1100 series model equipped with a photodiode array detector and a mass detector in series (Agilent Technologies, Waldbronn, Germany). The equipment consisted of a binary pump (model G1312A), an autosampler (model G1313A), a degasser (model G1322A), and a photodiode array detector (model G1315B). The HPLC system was controlled by ChemStation software (Agilent, version 08.03). The mass detector was an ion trap spectrometer (model G2445A) equipped with an electrospray ionization interface, and was controlled by LCMSD software (Agilent, version 4.1). The ionization conditions were 350 °C and 4 kV, for capillary temperature and voltage, respectively. The nebulizer pressure and nitrogen flow rate were 65.0 psi and 11 L/min, respectively. The full-scan mass covered the range of m/z from 100 to 1200. Collision-induced fragmentation experiments were performed in the ion trap using helium as the collision gas, with voltage ramping cycles from 0.3 to 2 V. The mass spectrometry data were acquired in the positive ionization mode for anthocyanins and in the negative ionization mode for other flavonoids. The MSn was carried out in the automatic mode on the more-abundant fragment ion in MS (n-1).

### Statistical analysis

To calculate cytotoxicity (CC_50_) and lytic plaques reduction (IC_50_) the GraphPadPrism program (Software, San Diego, CA.) was used. The selectivity index (SI) was calculated by the ratio of the value of CC_50_/IC_50_.

## Results

### Obtaining the plant extracts

Extracts of four plant species at three distinct temperatures using two solvents were obtained. Each extract was performed in triplicate; therefore, 72 final individual extracts were evaluated.

### Cytotoxicity of plant extracts

The cytotoxicity (CC_50_) values were in the range of 200 to 1100 μg/mL. The most toxic extract was of *C. pulcherrima* obtained with methanol at 60 °C (241.03 ± 9.93 μg/mL), while the less toxic was of *T. officinale* obtained with methanol at 120 °C (1,013.45 ± 33.84 μg/mL). Results for all extracts are shown in Tables 1 and 2. Considering the cytotoxic potential, working concentrations below the CC_50_ were defined to evaluate the antiviral activity.

**Table 1:** Cytotoxic concentration 50 (CC_50_) of the aqueous extracts to 60, 85 and 120 °C

	CC_50_ (μg/ml)		
	60 °C	85 °C	120 °C
*T. officinale*	617.26 ± 23.62	762.50 ± 27.79	675.63 ± 29.80
*U. dioica*	373.78 ± 22.77	448.68 ± 26.99	493.43 ± 10.26
*C. integrifolia*	876.18 ± 29.62	926.31 ± 40.89	991.47 ± 35.00
*C. pulcherrima*	719.07 ± 23.10	904.56 ± 23.54	941.42 ± 29.10

**Table 2:** Cytotoxic concentration 50 (CC_50_) of the methanolic extracts to 60, 85 and 120 °C

	CC_50_ (μg/ml)		
	60 °C	85 °C	120 °C
*T. officinale*	968.38 ± 37.17	891.92 ± 29.19	1013.45 ± 33.84
*U. dioica*	702.50 ± 14.50	669.15 ± 17.13	830.07 ± 18.20
*C. integrifolia*	758.78 ± 20.33	715.02 ± 16.17	745.18 ± 21.88
*C. pulcherrima*	241.03 ± 9.93	325.09 ± 14.34	344.17 ± 13.24

### The aqueous extracts have low potential to inhibit the viral infection

The inhibitory activity on DENV2 infection in cell culture was different with each of the extracts, which were tested between 25 and 200 μg/mL. The aqueous extracts showed little inhibition potential, for example, *C. pulcherrima*, *U. dioica* and *C. integrifolia* decreased the infection level between 5 and 10% at 200 μg/mL. The aqueous extract with higher activity against DENV was obtained for *T. officinale* at 60 °C, which caused 53% inhibition at the maximum tested concentration (200 μg/mL). None of the other extracts decreased more than 50% the number of lytic plaques produced by the virus (data not shown). None of the aqueous extracts were used in subsequent studies.

### Methanolic extracts of *T. officinale* and *U. dioica* inhibit the replication of DENV2

**Fig. 1:**
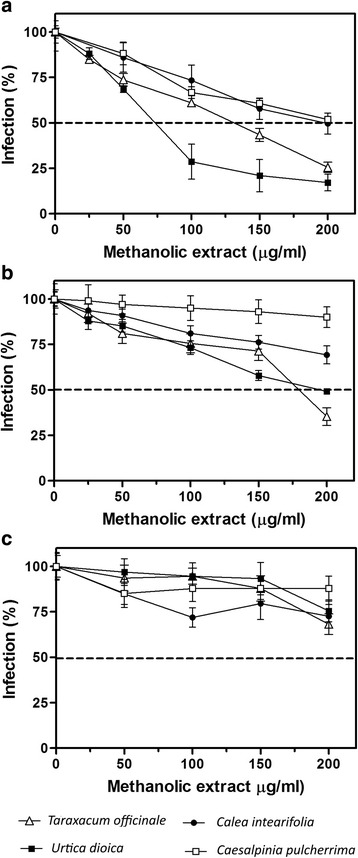
Effect of methanolic plant extracts on the DENV replication in cell culture. Methanolic extracts at 60 (**a**), 85 (**b**) and 120 °C (**c**) of *U. dioica*, *T. officinale*, *C. integrifolia* and *C. pulcherrima* were tested in order to inhibit the replication in vitro of DENV2

**Fig. 2:**
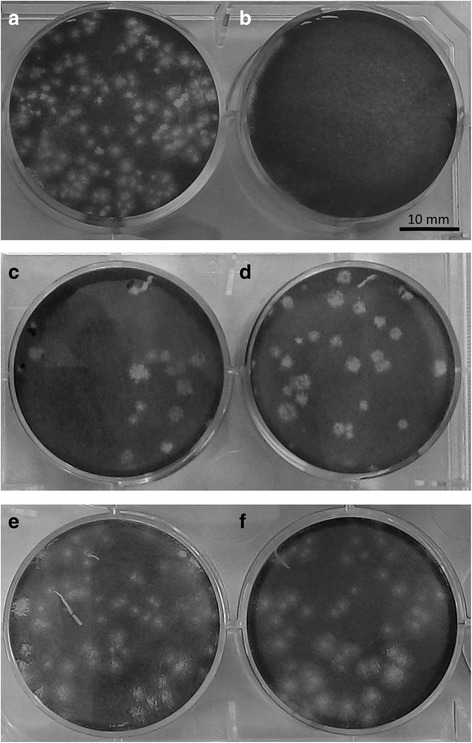
Inhibiting the formation of lytic plaques using methanolic extracts at 60 °C. DENV infection in BHK21 cells were inhibited incubating the plant extracts with every extract before the infection assay. Effect of 200 μg/ml of *U. dioica* (**c**), *T. officinale* (**d**), *C. integrifolia* (**e**) and *C. pulcherrima* (**f**) extracts are shown. Non-inhibited and non-infected controls are shown in **a** and **b**, respectively

The methanolic extracts at 60 °C of all the four plants, inhibited the viral replication in a dose-dependent manner. However, only *T. officinale* and *U. dioica* extracts reach more than 50% inhibition of the lytic plaques, therefore only for them the IC_50_ was calculated and were 132.50 ± 3.25 and 74.5 ± 1.70 μg/ml, respectively (the SI values were 7.31 and 9.43 for each extract) (Table 3, Fig. 1). Figure 2 shows some examples of the inhibition of lytic plaques with methanolic extracts at 60 °C of the four tested plants.

**Table 3:** Half maximal inhibitory concentration (IC_50_) and selectivity index (SI) on DENV2 replication of methanolic extracts at 60 and 85 °C and the *T. officinale* and *U. dioica* fractions obtained by silica gel chromatography

	IC_50_ (μg/ml)	CC_50_ (μg/ml)	SI
*T. officinale* (60 °C)	132.50 ± 3.25	968.38 ± 37.17	7.31
*U. dioica* (60 °C)	74.51 ± 1.70	702.50 ± 14.50	9.43
*T. officinale* (85 °C)	179.1 ± 6.56	891.92 ± 29.19	4.98
*U. dioica* (85 °C)	194.7 ± 3.63	669.15 ± 17.13	3.44
*T. officinale* F-09^a^	165.70 ± 3.85	925.45 ± 29.27	5.59
*U. dioica* F-07^a^	126.10 ± 2.80	758.25 ± 24.75	6.01

SI was calculated by the formula: CC_50_/IC_50_

^a^Fraction number obtained by chromatography

The extracts obtained at 85 °C showed also inhibition properties (Fig. 1b) and the IC_50_ values were calculated as 179.1 ± 6.56 and 194.7 ± 3.65 μg/mL for *T. officinale* and *U. dioica*, respectively (Table 3, Fig. 1). The rest of the extracts obtained at 85 °C decreased the viral infection only slightly. The extracts obtained at 120 °C of the four plants showed no consistent inhibitory effect on the formation of lytic plaques (Fig. 1c).

### Antiviral activity of *T. officinale* and *U. dioica* extracts fractioned by silica gel chromatography

**Fig. 3:**
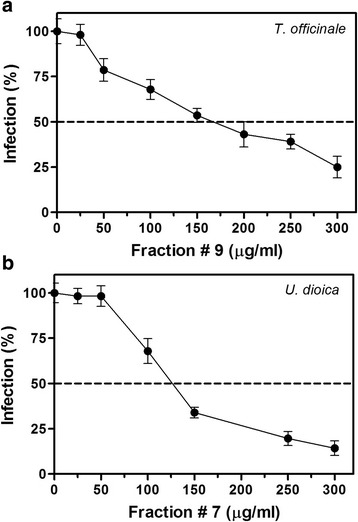
Antiviral activity of fractions 7 and 9 of *T. officinale* (**a**) and *U. dioica* (**b**) extracts obtained by silica gel chromatography

Extracts of *T. officinale* and *U. dioica* obtained with methanol at 60 °C were fractioned. Seventeen fractions for every extract were obtained. All fractions were tested to identify antiviral activity and to quantify their cytotoxicity. The higher antiviral activity was identified in the fractions (F) 9 and 7 of *T. officinale* and *U. dioica*, respectively. In separate assays, both F9 and F7 inhibited the DENV2 replication in a dose-dependent manner. The calculated IC_50_ value for *T. officinale* was 165.70 ± 3.85 and for *U. dioica* was 126.10 ± 2.80 μg/ml, while SI values were 5.59 and 6.01, respectively (Table 3, Fig. 3).

### Compounds identified in *T. officinale* F9

Analysis of *T. officinale* F9 showed major peaks of phenolic metabolites that have been previously reported in literature and were characterized by HPLC-PDA-ESI-MS/MS (negative ionization mode) as presented in Table 4, besides other compounds present in lesser amounts.

**Table 4:** Compounds identified in *T. officinale* fraction 9 by HPLC-PDA-ESI-MS/MS

Compound		tR, min	UVmax, nm	[M-H], m/z	MS2 – MS3
					Fragment ions, m/z
1	Caffeoyl hexoside	13.5	290	341	179, 135
2	5-O-Caffeoylquinic acid	20.3	326	353	191
3	Caffeic acid	23.5	322	179	135
4	Quercetin diglycoside	25.2	358	595	463, 301
5	Quercetin diglycoside	29.9	358	595	463, 301
6	Luteolin diglycoside	38.6	338	609	285
7	Quercetin-3-O-rutinoside	45.4	358	609	301
8	Luteolin-7-O-rutinoside	46.1	348	593	285
9	Luteolin-7-O-glucoside	47.5	346	447	285
10	Chicoric acid	48.4	328	473	311, 179
11	Chicoric acid	51.7	328	473	311, 179, 149
12	Luteolin-7-O-rutinoside	53.2	348	593	285
13	3,5-di-O-Caffeoylquinic acid	54.1	325	515	353, 191
14	4,5-di-O-Caffeoylquinic acid	60.0	327	515	353, 179, 173

The compound 1 was characterized as caffeoyl hexoside (m/z 341) which showed the loss of a saccharide moiety in the MS2 (162 Da). The caffeoylquinic acid (CQA) was characterized by means of –MS2 of its deprotonated molecular ion (m/z 353) giving a base peak at m/z 191, and the m/z 179 ion is weak or undetectable, and according to Clifford et al., [38] can be labeled as 5-caffeoylquinic acid (5-CQA) (2). Additionally, caffeic acid (3) and di-caffeoylquinic acids (di-CQA) ([M-H]-, m/z 515) were also present (13 and 14).

Besides caffeoylquinic acids, two dicaffeoyltartaric acid derivatives were detected. Both components (10 and 11), showed [M-H] ion at m/z 473 and the formation of a predominant product ion at m/z 311 (loss of a caffeoyl moiety). In the MS2 and MS3 experiment the loss of a second caffeic acid moiety was observed, resulting in a base peak at m/z 149. Since this fragmentation was already reported, it is concluded that these compounds are chicoric acids, also present in lettuces [39].

With respect to the flavonoids, in the UV chromatogram hydroalcoholic extracts we have detected seven compounds (4 – 9 and 12). The compounds 4 and 5 provided [M-H] ions at m/z 595, and MS2 and MS3 fragmentations for a quercertin diglycoside with a pentose and a hexose moiety linked in distinct positions of the aglycone (m/z 463, 301).

The compound 7 showed UV spectra of quercetin derivative [40] and deprotonated molecular ion at m/z 609. Its MS2 fragmentation gave the unique peak at m/z 301 corresponding to the deprotonated ion of its aglycon, as well as absence of intermediate ions, indicating a interglycosidic linkage rhamnosyl(1 → 6)glucoside, (rutinoside) [41]. All these data indicate that this compound is quercetin-3-O-rutinoside (rutin), widely distributed in plants. F9 also presented several luteolin glycosides (6, 8, 9, and 12), showing a prominent fragment at m/z 285. The order of elution and identities were assigned according to the previous reports of the presence of these compounds in dandelion [39].

### Compounds identified in *U. dioica* F7

According to the results of the LC-MS analyses and major fragments in MS2 (ESI negative mode), there were not any gallic acid, syringic, fumaric, vanillic, isorhamnetin or flavanols in the samples, but there were chlorogenic acid (353 m/z), rutin (609 m/z), and flavonol glycosides (e.g. quercetin and kaempferol glycosides, with 463 and 447 m/z, respectively) (Table 5).

**Table 5:** Compounds identified in *U. dioica* fraction 7 by HPLC-PDA-ESI-MS/MS

Compound		tR, min	UVmax, nm	[M-H], m/z	MS2 m/z
1	Coumaroyl derivative	15.6	320	387	163
2	Sinapoyl derivative	22.5	322	433	223
3	Quercetin-3-*O*-rutinoside	43.1	352	609	301
4	Quercetin-3-*O*-hexose	45.6	352	463	301
5	Kaempferol-3-*O*-hexose	45.7	350	447	285
6	Kaempferitrin	49.6	356	577	285
7	Kaempferol-3-*O*-hexose	54.0	348	447	285

## Discussion

Multiple studies have reported the discovery or obtaining of compounds with anti-dengue properties [8, 16–18, 29, 42–47], however, up to now there are no commercially available drugs for treatment of dengue.

In the present study we included plants widely used in traditional medicine in many countries for the treatment of many diseases or organic disorders [48, 49]. Extracts from our selected plants presented different activities per species, temperature and solvent used. The lesser toxic extract was *T. officinale* obtained with methanol and the more toxic was the methanolic extract obtained from *C. pulcherrima*.

We performed a protocol in which the cells were infected and subsequently, the plant extract was added as possible inhibitor, trying to simulate a system with an active DENV infection, like it occurs in a natural infection. Therefore, this protocol suggests that the inhibitory effect of virus replication is obstructed in an intermediate step in the replication cycle, similar to what has been reported in other experimental inhibition of DENV [18], possibly by interaction of flavonoids with viral enzymes involved in the ARN synthesis or maturation of polyprotein.

By comparing the extracts obtained at three different temperatures, we observed a higher inhibition of DENV2 replication with those obtained at 60 °C (Fig. 1a), suggesting the selection and conservation of compounds with anti-dengue properties. The time and temperature of extraction of compounds with pharmacological potential are important parameters to optimize production and lower costs for the overall process. Different authors propose that increasing the temperature in the extraction process promotes increased solubility and the diffusion coefficient of phenolic compounds, provided they do not cause denaturation [50, 51].

A similar process occurs with *C. pulcherrima,* the aqueous extracts of this plant contain compounds like quercetin with a broad inhibitory activity on adenovirus 8 and 3 and on herpesviruses [26]. It is possible that the reason for the activity is not a single molecule, but a mixture of them and that could have an effect on different viruses through different mechanisms.

An important parameter to consider is the selectivity index (SI), which ranged from 5.59 and 9.43 for the extracts or fractions. These values are similar to other reports of natural products with some anti-dengue activity [18, 19, 47, 52, 53]. SI represents the relative effectiveness of a product in inhibiting viral replication compared to inducing cell citotoxicity. High therapeutic index represents a relative low cytotoxicity and high antiviral activity. This parameter is used for in vitro tests, unlike the therapeutic index which is a measurement with a similar meaning, but performed in vivo [54, 55].

In our work the aqueous extracts showed less inhibition compared with extracts obtained with methanol, as it was reported previously [42] to assess the inhibitory effect of aqueous and methanol extracts of *Hydrocotyle sibthorpioides* on the replication of DENV.

Through HPLC-DPA-ESI-MS/MS we identified 11 compounds in the *T. officinale* F9 and 6 in the *U. dioica* F7. In both fractions, quercetin derivatives were found. Several studies report that quercetin have anti-dengue activity [18, 43, 44], although the specific mechanisms remain undetermined. However, the interaction of quercetin or its derivatives with the NS2B-NS3 protease [45, 56], the envelope (E) protein [57, 58] and the NS5 polymerase [59] have been predicted by in silico methods.

On the other hand, luteolin derivatives were found in the *T. officinale* fraction. Luteolin has been reported to have inhibitory activity on several viruses such as enterovirus 71, Coxsackievirus A16 [60] and chikungunya virus [61]. In recent articles, the interaction of one of the identified compounds, luteolin-7-O-glucoside, and other luteolin derivatives, has been predicted to interact with the NS2/NS3 protease using in silico analysis [46, 56]. In addition, a very recent study showed that luteolin reduces DENV infection through the inhibition of human furin, which is an enzyme involved in the maturation of the virions, whereby viral particles with a low infectious capacity are produced [53].

Other compounds identified in *T. officinale* F9 were caffeic acid and caffeoylquinic acid derivatives. These molecules have been shown as inhibitors of some important viruses such as hepatitis B [62], influenza A [63], herpes simplex [64] and more recently, dengue virus [47]. In this last study, Zanello et al., show the effect of two derivatives on the four DENV serotypes.

## Conclusions

It is interesting that analyzed fractions of *T. officinale* and *U. dioica* have several molecules with demonstrated antiviral activity. Recently, an antiretroviral activity (HIV-1) in aqueous extracts of *T. officinale* was identified [24], while other studies have identified an inhibitory effect against yellow fever virus (prototype of *Flaviviridae*) [65]. Is it possible that different compounds in this plant could have effects against different viruses?

The study of the recognized components present in our factions is determinant to identify the probable anti-dengue molecule and/or their effects on other viruses, including the recent mosquito-borne emerging viruses chikungunya and Zika. It is also necessary to study a formulation of several active compounds in these fractions, searching a probable synergism in the process of inhibition of virus infection. Our group is currently performing these studies.
